# Antioxidant and Antiproliferative Activities of Twenty-Four *Vitis vinifera* Grapes

**DOI:** 10.1371/journal.pone.0105146

**Published:** 2014-08-18

**Authors:** Zhenchang Liang, Lailiang Cheng, Gan-Yuan Zhong, Rui Hai Liu

**Affiliations:** 1 Institute of Botany, the Chinese Academy of Sciences, Beijing, China; 2 Department of Food Science, Cornell University, Ithaca, New York, United States of America; 3 Department of Horticulture, Cornell University, Ithaca, New York, United States of America; 4 USDA -ARS Grape Genetics Research Unit, Geneva, New York, United States of America; University of Sassari, Italy

## Abstract

Grapes are rich in phytochemicals with many proven health benefits. Phenolic profiles, antioxidant and antiproliferative activities of twenty-four selected *Vitis vinifera* grape cultivars were investigated in this study. Large ranges of variation were found in these cultivars for the contents of total phenolics (95.3 to 686.5 mg/100 g) and flavonoids (94.7 to 1055 mg/100 g) and antioxidant activities (oxygen radical absorbance capacity 378.7 to 3386.0 mg of Trolox equivalents/100 g and peroxylradical scavenging capacity14.2 to 557 mg of vitamin C equivalents/100 g), cellular antioxidant activities (3.9 to 139.9 *µ*mol of quercetin equivalents/100 g without PBS wash and 1.4 to 95.8 *µ*mol of quercetin equivalents /100 g with PBS wash) and antiproliferative activities (25 to 82% at the concentrations of 100 mg/mL extracts).The total antioxidant activities were significantly correlated with the total phenolics and flavonoids. However, no significant correlations were found between antiproliferative activities and total phenolics or total flavonoids content. Wine grapes and color grapes showed much higher levels of phytochemicals and antioxidant activities than table grapes and green/yellow grapes. Several germplasm accessions with much high contents of phenolics and flavonoids, and total antioxidant activity were identified. These germplasm can be valuable sources of genes for breeding grape cultivars with better nutritional qualities of wine and table grapes in the future.

## Introduction

Fruits, as an important part of diet, are beneficial for human health.The Dietary Guidelines for Americans provide three reasons to eat more fruits and vegetables [1]. First, most fruits and vegetables are major contributors of a number of nutrients that are under-consumed in the United States, including folate, magnesium, potassium, dietary fiber, and vitamins A and C. Second, consumption of fruits and vegetables is associated with reduced risk of many chronic diseases. Specifically, moderate evidence indicates that intake of at least 2.5 cups of vegetables and fruits perday is associated with a reduced risk of cardiovascular disease, including heart attack and stroke. Some fruits and vegetables may be protective against certain types of cancer. Third, most fruits and vegetables, when prepared without added fats or sugars, are relatively low in calories. Consumption of fruits and vegetables instead of higher calorie foods can help adults and children achieve and maintain healthy weight. Currently, very few Americans consume diets that meet Dietary Guideline recommendations of fruits and vegetables. In fact, most Americans only consume about 42% of goal intake for fruits. For improving public health and reducing the risk of developing chronic diseases, the Dietary Guidelines for Americans recommend that most people should eat at least 9 servings of fruits and vegetables per day based on a 2000 kcal diet [1].

Grapes (*Vitis sp*.) are one of the most important fruit crops worldwide. They are consumed as fresh fruits as well as wine, juice and other processed products. There are about 60 grape species in the genus of *Vitis*, and the species *V. vinifera*, or European grapes, is most widely cultivated. There are thousands of *V. vinifera* cultivars extant in the world. To obtain an assessment of the full range of phytochemicals in grapes, we recently analyzed 36 phenolic compounds in the berry samples of 344 representative *V. vinifera* cultivars [2] and supported previous findings that grapes are rich in phytochemicals [3], [4]. Demonstration of health benefits of these phytochemicals in grapes has been actively pursued. It has been reported that grape extracts exhibited antioxidant activities, including scavenging of free radicals, inhibition of lipid oxidation, and reduction of hydroperoxide formation [4], [5], and inhibit cardiovascular diseases and certain types of cancers, reducing plasma oxidative stress, and slowing aging [6], [7], [8].While there were many reports on the assessment of phytochemical compounds and their antioxidant activities of grapes, these studies were limited to only a few cultivars [9]–[15].

In this study, we determined the phytochemical profiles of 24 *V. vinifera* grape cultivars and analyzed their total antioxidant and antiproliferative activities. These 24 cultivars were selected on the basis of a previous study in which we demonstrated that these cultivars contained a wide range of the composition and content of phenolic compounds [2]. The contribution of flavonoids to total phenolics and correlative relationships among phenolics, flavonoids, antioxidant activities and antiproliferative activities were also investigated.

## Materials and Methods

### Chemicals and reagents

Methanol (MeOH), ethanol (EtOH), acetone, hexane, ethyl acetate, hydrochloric acid (HCI), acetic acid (HAC), potassium chloride (KCI), sodium acetate (NaAC), sodium carbonate (NaCO3), sodium hydroxide (NaOH), potassium phosphate monobasic (KH_2_PO4), and potassium phosphate dibasic (K_2_HPO4) were of analytical grade and purchased from Mallinckrodt Chemicals (Phillipsburg, NJ). Ascorbic acid, 2′, 7′-dichlorofluorescin diaacetate (DCFH-DA), fluorescein disodium salt, sodium borohydride (NaBH_4_, reagent grade), chloranil (analytical grade), vanillin (analytical grade), quercetin dehydrated, catechin hydrated, Folin-Ciocalteu reagent, 6-hydroxy-2, 5, 7, 8-tetramethylchroman-2-carboxylic acid (Trolox), trifluoroacetic acid (TFA, chromatographic grade), and acetonitrile (chromatographic grade) were purchased from Sigma-Aldrich, Inc. (St. Louis, MO). Tetrahydrofuran (THF, analytical grade), dimethyl sulfoxideand aluminum chloride (AlCl_3_•6H_2_O, analytical grade) were purchased from Fisher Scientific (Fair Lawn, NJ). Gallic acid was obtained from ICN Biomedicals, Inc. (Aurora, OH). 2, 2′-Azobis (2-amidinopropane) dihydrochloride (ABAP) was purchased from Wako Chemicals USA, Inc. (Richmond, VA). HepG2 liver cancer cells were obtained from the American Type Culture Collection (ATCC) (Rockville, MD). Williams' Medium E (WME) and Hanks' Balanced Salt Solution (HBSS) were purchased from Gibco Life Technologies (Grand Island, NY). Fetal bovine serum (FBS) was obtained from Atlanta Biologicals (Lawrenceville, GA).

### Plant materials

Berry samples of 24 *V. vinifera* grape cultivars were collected from the United States Department of Agriculture-Agricultural Research Service (USDA-ARS) *Vitis* clonal repository in Davis, California (Table 1). The grape berries were harvested upon ripening from the 2010 vintages. All the vines received standard fertilization, irrigation, pruning, and insect and disease control. About 100 of representative berries were collected from each individual cultivar. Berry color was scored as green-yellow, pink, red, dark-red violet, blue black or red-black and compared with those recorded in the Germplasm Resources Information Network [16] when a cultivar was introduced. The number of berries was counted and the berry weight was recorded for each sample before being frozen and stored at −80°C for further processing. The frozen berries were then crushed using a mortar and pestle. After removing all the seeds, flesh and peel tissues were ground in an IKA A11 mill (IKA Works, Inc, NC, USA) into powder while frozen using liquid nitrogen. All data collected for each grape cultivar were reported as mean ± SD with at least three replications.

**Table 1:** Description of *V. vinifera* grape samples used in the study and total phenolics (µmol of Gallic acid equiv /100 g FW) and flavonoids (µmol of Catechinequiv /100 g FW) content, ratio of total flavonoids to total phenolics (F/P, %), ORAC (mg of Trolox equivalents/100 g FW) and PSC (mg of vitamin C equivalents/100 g FW) values, CAA with PBS washing and no PBS washing (µmol of QE/100 g of grape) values.

Accession	Cultivar	Primary use	Color	Phenolics	Flavonoids	F/P	ORAC	PSC	CAA with wash	CAA with no wash
DVIT2630	Agwam	Wine grape	Red	102.7±3.0	128.6±8.6	73.5±5.2	547.3±50.5	73.5±7.5	1.4±0.2	5.3±0.3
DVIT0632	Araclinos	Wine grape	Blue-black	347.9±7.1	408.1±11.2	68.9±2.7	1171.3±58.3	232.1±19.9	24.6±1.0	33.9±2.1
DVIT0677	Cabernet Sauvignon	Wine grape	Red-black	448.3±5.2	603.1±34.5	79.0±5.1	1394.7±54.3	226.0±18.0	22.0±2.6	43.6±5.3
DVIT0696	Corbeau	Wine grape	Red-black	582.8±11.7	687.1±43.5	69.0±3.5	2320.0±129.0	420.5±30.8	30.7±1.2	46.1±3.3
DVIT0384	Coudsi	Table grape	Green-yellow	159.5±4.8	201.7±14.4	74.0±3.2	710.3±63.6	31.2±1.4	9.8±1.2	14.7±1.5
DVIT0314	Demir Kara	Table grape	Red-black	345.0±13.2	341.0±13.3	58.0±1.0	1400.7±130.0	167.3±6.6	25.5±2.5	59.7±3.0
DVIT2705	Dornfelder	Table grape	Blue-black	391.6±9.5	530.0±36.4	79.3±4.7	1479.7±149.0	404.5±18.1	49.6±5.7	54.6±3.2
DVIT2642	Madam Matijas	Table grape	Green-yellow	201.5±5.4	146.4±7.8	42.6±2.4	566.0±67.0	73.0±6.5	10.2±0.9	12.2±0.1
DVIT0099	Mathilde	Table grape	Red	201.9±9.1	170.8±11.3	49.6±5.1	630.7±14.0	109.3±10.8	3.4±0.3	8.0±1.0
DVIT0825	Melon	Wine grape	Green-yellow	466.8±9.9	587.5±16.0	73.1±2.9	1651.7±138.0	103.8±10.9	11.9±0.8	42.5±0.6
DVIT1042	Mermark	Wine grape	Red-black	664.4±18.2	568.0±21.7	50.1±0.8	2422.7±221.0	244.0±15.6	14.1±1.3	47.5±1.5
DVIT0465	Muscat of Alexandria	Table grape	Green-yellow	95.3±1.8	94.7±3.0	58.3±1.2	392.0±34.9	14.2±0.6	1.8±0.2	3.9±0.1
DVIT2646	Pearl of Zola	Table grape	Green-yellow	291.2±1.5	230.0±4.1	46.3±0.7	733.0±65.2	58.0±3.5	17.0±1.2	26.4±0.9
DVIT2700	Pirobelle	Table grape	Blue-black	207.8±10.5	210.6±15.2	59.4±2.5	571.0±37.7	117.6±3.7	21.2±0.3	23.1±0.7
DVIT1119	Plavac Mali	Wine grape	Blue-black	351.1±11.0	502.6±31.8	84.2±6.5	1186.0±122.0	138.2±8.8	33.0±1.8	34.3±1.5
DVIT0503	Ribier	Table grape	Blue-black	294.3±7.9	251.9±10.1	50.2±2.6	909.3±93.5	46.9±6.0	19.9±2.5	21.5±2.6
DVIT0934	Robola	Wine grape	Green-yellow	384.4±13.6	425.2±27.4	65.1±5.2	1298.3±135.0	91.5±7.3	14.5±0.5	47.4±0.2
DVIT0937	Royalty	Wine grape	Red-black	686.5±1.9	1054.6±78.1	90.0±6.8	3386.3±221.0	557.0±50.0	95.8±5.0	139.9±9.2
DVIT0940	Ruby Cabernet	Wine grape	Red-black	486.7±13.2	758.2±48.1	91.3±3.9	1752.3±174.0	183.7±16.3	73.3±7.2	79.8±4.7
DVIT1060	Saint Macaire	Wine grape	Red-black	368.2±17.6	301.2±19.1	47.9±5.1	1402.7±158.0	285.0±26.6	16.9±1.0	50.0±2.9
DVIT0944	Salvador	Wine grape	Red-black	407.5±8.9	485.6±30.8	69.8±3.4	2504.7±271.0	376.2±36.7	81.1±7.7	91.0±8.8
DVIT0535	Thompson Seedless	Table grape	Green-yellow	151.1±6.6	201.4±7.3	78.3±3.2	378.7±24.8	24.1±2.2	7.0±0.6	10.5±0.9
DVIT1061	Touriga	Wine grape	Red-black	482.5±20.8	331.5±19.8	40.3±3.6	1960.3±85.6	302.0±11.0	21.1±0.1	54.4±2.3
DVIT0605	Yourutico	Table grape	Blue-black	419.1±6.0	534.6±29.3	74.7±3.8	1365.0±74.7	134.9±7.3	11.4±0.9	45.9±3.6

### Extraction of total phenolic compounds

Total phenolics were extracted from ground berry samples using the method previously reported from our laboratory [17]. Briefly, 10 g of grape powder was homogenized with 30 mL of 80% chilled acetone/water using a Virtis High Speed Homogenizer for 3 min on the ice. After centrifugation at 3000 g for10 min at 4°C, the supernatant was collected, and 30 mL of fresh extraction solution were added and the extraction was repeated twice. All extracts were transferred into a round bottom flask, and were evaporated using a rotary evaporator at 45°C until the weight of the evaporated filtrate was less than10% of the weight of the original filtrate. The extracts were brought to a final volume of 10 mL with 70% methanol. All extracts were stored at −40°C until use. All extractions were analyzed in triplicates.

### Determination of total phenolic contents

Total phenolic content was determined using the Folin-Ciocalteu colorimetric method [18], which was modified by our laboratory [19]. Briefly, all extracts were diluted 1∶20 with distilled water to obtain readings within the standard curve ranges of 0.0–600.0 µg of gallic acid/mL. The grape extracts were oxidized by the Folin-Ciocalteu reagent and the reaction was acted in sodium carbonate. The absorbance was measured at 760 nm, after 90 min at room temperature by a MRX II Dynex plate reader (Dynex Technologies Inc., Chanilly, VA). The absorbance values were then compared with those of standards with known gallic acid concentrations. All values were expressed as the mean (milligrams of gallic acid equivalents per 100 g of fresh sample) ± SD of three replications.

### Determination of total flavonoid contents

The total flavonoid content was determined using the sodium borohydride/chloranil-based (SBC) assay developed by our laboratory [20] with modifications [21]. Briefly, 0.2 mL extracts of tested samples were added into test tubes (15×150 mm), dried under nitrogen gas, and reconstituted in 1 mL of terahydrofuran/ethanol (THF/EtOH, 1∶1, v/v). Catechin standards (0.1–10.0 mM) were prepared fresh before use in 1 mL of THF/EtOH (1∶1, v/v). Then 1 mL of 50 mM NaBH_4_ solution and 0.5 mL of 74.6 mM AlCl_3_ solution were added into each test tube with 1 mL of sample solution or 1 mL of catechin standard solution. The test tubes were shaken in an orbital shaker at room temperature for 30 min. An additional 0.5 mL of 50.0 mM NaBH_4_ solution was added into each test tube with shaking continued for another 30 min at room temperature. Two millilitersof cold 0.8 M acetic acid solution was added into each test tube, and the solution was kept in the dark for 15 min after a thorough mix. One milliliter of 20.0 mM chloranil was added into each tube, which was heated at 95 °C with shaking for 60 min. The reaction solution was cooled using tap water, and the final volume was brought to 4 mL using methanol. One milliliter of 1052 mM vanillin was added into each tube and mixed. Then 2 mL of 12 M HCl was added to each tube, and the reaction solution was kept in the dark for 15 min after a thorough mix. Aliquots of final reaction solutions (200 µL) were added into each well of a 96-well plate, and the absorbance was measured at 490 nm using a MRX Microplate Reader with Revelation workstation (Dynex Technologies, Inc., Chantilly, VA). Total flavonoid content was expressed as milligrams of catechin equivalents per 100 g of fresh weight of sample. Data were reported as mean ± SD with at least triplicates.

### Oxygen radical absorbance capacity (ORAC) assay

The antioxidant activity was determined using oxygen radical absorbance capacity (ORAC) assay [22] and modified in our laboratory [23]. Phenolic extract dilutions were prepared with 75 mM phosphate buffer (pH 7.4). The assay was performed in black-walled 96-well plates (Corning Scientific, Corning, NY). The outside wells of the plate were not used as there was much more variation from them than from the inner wells. Each well contained 20 µL extracts or 20 µLTrolox standard (range 6.25–50 µM), and 200 µL fluoroscein (final concentration 0.96 µM), which were incubated at 37°C for 20 min. After incubation, 20 µL of 119 mM ABAP was added to each well. Fluorescence intensity was measured using Fluoroskan Ascent FL plate-reader (Thermo Labsystems, Franklin, MA) at excitation of 485 nm and emission of 520 nm for 35 cycles every 5 min. ORAC values were expressed as milligrams of TE/100 g FW. Data were reported as mean ± SD with triplicates.

### Peroxylradicalscavenging capacity (PSC) assay

The antioxidant activity of extracts was determined by using a PSC assay developed in our lab [24] with modifications [25]. Just prior to use in the reaction, 107 µL of 2.48 mM dichlorofluorescein diacetate was hydrolyzed to dichlorofluorescein with 893 µL of 1.0 mM KOH for 5 min in a vial to remove the diacetate moiety and then diluted with 7 mL of 75 mM phosphate buffer (pH 7.4). ABAP (200 mM) was prepared fresh in the buffer and was kept at 4°C between runs. In an assay, 100 µL of extracts was diluted in 75 mM phosphate buffer (pH 7.4) and then transferred into reaction cells on a 96-well plate, and 100 µL of dichlorofluorescein was added. The 96-well plate was loaded into a Fluoroskan Ascent fluorescence spectrophotometer (Thermo Labsystems, Franklin, MA), and the solution in each cell was mixed by shaking at 1200 rpm for 20 s. The reaction was then initiated by adding 50 µL of ABAP from the autodispenser on the equipment. Each set of dilutions for a replicate and control was analyzed three times in adjacent columns. The reaction was carried out at 37°C, and fluorescence was monitored at 485 nm excitation and 538 nm emission with the fluorescence spectrophotometer. The buffer was used for control reactions. Data were acquired with Ascent software, version 2.6 (Thermo Labsystems). Data were reported as mean ± SD with three replicates.

### Cell Culture

HepG2 cells (The American Type Culture Collection, ATCC, Rockville,MD) were grown in growth medium (WME supplemented with 5% FBS, 10 mMHepes, 2 mM L-glutamine, 5 *µ*g/mL insulin, 0.05 *µ*g/mL hydrocortisone, 50 units/mL penicillin, 50 *µ*g/mL streptomycin, and 100 *µ*g/mL gentamicin) and were maintained at 37°C and 5% CO_2_ as described previously [26], [27]. Cells used in this study were between passages or generations18 and 28 at which the cells were at the most stable physiological status.

### Cytotoxicity

Cytotoxicity was measured using the methylene blue assay developed by our laboratory (Felice et al., 2009) with modifications [28]. Briefly, HepG2 cells were seeded at 4×10^4^/well on a 96-well plate in 100 *µ*L of growth medium and incubated for 24 h at 37°C. The medium was removed, and the cells were washed with PBS. Treatments of fruit extracts or antioxidant compounds in 100 *µ*L of treatment medium (WME supplemented with 2 mM L-glutamine and 10 mM Hepes) were applied to the cells, and the plates were incubated at 37°C for 24 h. The treatment medium was removed, and the cells were washed with PBS. A volume of 50 *µ*L/well methylene blue staining solution (98% HBSS, 0.67% glutaraldehyde, 0.6% methylene blue) was applied to each well, and the plate was incubated at 37°C for 1 h. The dye was removed, and the plate was immersed in fresh deionized water six times, or until the water became clear. The water was tapped out of the wells, and the plate was allowed to air-dry briefly before 100 *µ*L of elution solution (49% PBS, 50% ethanol, 1% acetic acid) was added to each well. The microplate was placed on a bench-top shaker for 20 min to allow uniform elution. The absorbance was read at 570 nm with blank subtraction using the MRX II DYNEX spectrophotometer (DynexInc., Chantilly, VA). Concentrations of fruit extracts that decreased the absorbance by >10% when compared to the control were considered to be cytotoxic (Felice et al., 2009).

### Cellular Antioxidant Activity (CAA) of grape extracts

The CAA was determined using the method previously developed in our laboratory [29]. Briefly, HepG2 cells were seeded at a density of 6×10^4^/well on a 96-well microplate in 100 *µ*L of growth medium/well. Twenty-four hours after seeding, the growth medium was removed, and the wells were washed with PBS. Wells were then treated in triplicate for 1 h with 100 *µ*L of treatment medium containing tested fruit extracts plus 25 *µ*M DCFH-DA. When a PBS wash was utilized, wells were washed with 100 *µ*L of PBS. When no PBS wash was done between treatments, the activity may have been higher, but the CV also tended to be higher. This was likely due to the interaction of the samples and oxidants with other factors in the residual medium on the cells. Washing the cells with PBS removed most of the interfering medium components and increased the consistency of the results. Then 600 *µ*M ABAP was applied to the cells in 100 *µ*L of HBSS, and the 96- well microplate was placed into a Fluoroskan Ascent FL plate reader (ThermoLabsystems, Franklin, MA) at 37°C. Emission at 538 nm was measured after excitation at 485 nm every 5 min for 1 h.

After blank subtraction and subtraction of initial fluorescence values, the area under the curve for fluorescence versus time was integrated to calculate the CAA value at each concentration of fruit as [30]

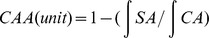
Where ∫SA is the integrated area under the sample fluorescence versus time curve and ∫CA is the integrated area from the control curve. The median effective dose (EC_50_) was determined for the fruits from the median effect plot of log(fa/*f*u) versus log(dose), where *f*a is the fraction affected (CAA unit) and *f*u is the fraction unaffected (1 - CAA unit) by the treatment. The EC_50_ values were stated as mean ± SD for triplicate sets of data obtained from the same experiment. EC_50_ values were converted to CAA values, expressed as micromoles of quercetin equivalents (QE) per 100 g of grape, using the mean EC_50_ value for quercetin from five separate experiments.

### Measurement of inhibition of HepG2 cell proliferation

The antiproliferative activity of different grape extracts was assessed by measurement of the inhibition of HepG_2_ (The American Type Culture Collection, ATCC, Rockville, MD) human cancer cell proliferation. Antiproliferative activities were determined by the colorimetric methylene blue method developed by our laboratory [31] with modifications [28], [32]. Human hepatocellular carcinoma HepG2 cells were seeded at a density of 2.5×10^4^/well on a 96-well microplate in 100 *µ*L of growth medium/well. The outside wells of the plate were not used as there was much more variation from them than those from the inner wells. After 4 h of incubation, the growth medium was removed and media containing various concentrations (10, 20, 40, 60, 80 and 100 mg/mL) of grape extracts were added to the cells. Control cultures received the extraction solution minus the grape extracts, and blank wells contained 100 µL of growth medium without cells. Cell proliferation (percent) was determined at 96 h from the absorbance reading at 570 nm for each concentration when compared to the control, using at least three replications for each sample. The effective median dose (EC_25_ and EC_50_) was determined and expressed as milligrams of grape extracts per milliliter ± SD.

### Statistical analysis

Data from this study were reported as mean ± SD with at least three replicates for each sample extract.All graphical representations were performed using Sigmaplot 10.0 for Windows (SPSS, USA), and all data were analyzed using SPSS (Statistics for Social Science) 13.0 for Windows. Results were subjected to ANOVA, and differences between means were located using Tukey B multiple comparison test. Correlations between various parameters were also investigated. Significance was determined at *P*<0.05.

## Results

### Total phenolic content

Table 1shows the total phenolic contents of 24 *V. vinifera* grape cultivars. Among all the grape cultivars analyzed, Royalty (red flesh cultivar) and Mermark had the highest total phenolic content (686.5±3.2 and 664.4±31.5 mg of gallic acid equivalents/100 g FW, respectively), and were significantly higher than other grape cultivars (*P*<0.05). Agwam and Muscat of Alexandria had the lowest total phenolic contents (*P*<0.05). There were 7-fold differences in the total phenolic contents between the highest (Royalty) and lowest (Muscat of Alexandria) ranked cultivars. Most green-yellow grape cultivars had lower total phenolic contents with no more than 300 mg of gallic acid equivalents/100 g FW except for Melon and Robola. On the contrary, colored grape cultivars had higher total phenolic contents and were more than 300 mg of gallic acid equivalents/100 g FW except for Ribier and two red cultivars of Mathilde and Agwam. All wine grape cultivars had higher total phenolic contents with more than 347.9 mg of gallic acid equivalents/100 g FW, but Agwam was an exception. All table grape cultivars had low phenolic contents except for Yourutico and Dornfelder.

### Total flavonoid content

Total flavonoid contents of the 24 grape cultivars are presented in Table 1. The Royalty grape presented the highest flavonoid content and was significantly higher than other cultivars (*P*<0.05). Compared to most other cultivars, Pearl of Zola, Pirobelle, Coudsi, Thompson Seedless, Mathilde, Madam Matijas, and Agwam had relatively lower levels of total flavonoid contents. Muscat of Alexandria had the lowest flavonoid content. There were almost 11-fold differences in total flavonoid contents between the highest and lowest ranked cultivars. Melon and Robola had higher total flavonoid contents than other green-yellow cultivars in which no significant differences were detected. In general, colored cultivars had higher total flavonoid contents than green-yellow cultivars (*P*<0.05). Compared to wine grape cultivars, all table grape cultivars had lower flavonoid contents (less than 250 mg of catechin equivalents/100 g FW) except for Yourutico, Dornfelder and Demir Kara. Total flavonoid contents of wine grape cultivars were higher than 300 mg of catechin equivalents/100 g FW except for Agwam.

### Contribution of total flavonoids to total phenolics

The contributions of total flavonoids to total phenolics, calculated on the basis of micromoles of the total flavonoids and total phenolics, are presented in Table 1. The contributions of total flavonoids to the total phenolicsin the 24 grape cultivars ranged from 40.3 to 91.3% with a mean 65.5%. The contributionsof flavonoids to total phenolics in Ruby Cabernet and Royalty (91.3 and 90% of the total phenolics, respectively) were the highest among all cultivars. In contrast, the total flavonoid contents were no more than 50% of the total phenolic contents in Mathilde, Saint Macaire, Pearl of Zola, Madam Matijas and Touriga.

### Oxygen radical absorbance capacity (ORAC)

The ORAC values for the 24 cultivars, expressed as mg of Trolox equivalents per 100 g of fresh grape, are shown in Table 1.Variation of ORAC values ranged from 378.7 (Thompson Seedless) to 3386.3 (Royalty) mg of Trolox equivalents/100 g FW, and the mean was 1339 mg. Royalty had the highest ORAC values, significantly higher than other cultivars (*P*<0.05). The other red flesh cultivars Salvador, Mermark and Corbeau also had relatively high ORAC values (*P*<0.05). Most green yellow cultivars had ORAC value no more than 733.0 mg of TE/100 g FW except for Melon and Robola. Most colored cultivars had ORAC values more than 909.0 mg of TE/100 g FW except for one blue-black cultivar (Pirobelle) and two red cultivars (Mathilde and Agwam). All table grape cultivars had lower ORAC values (lower than 1000 mg of TE/100 g FW) except for Dornfelder, Demir Kara and Yourutico. All ORAC values of wine grape cultivars were higher than 1170.0 mg of TE/100 g FW except for Agwam.

### Peroxyl radical scavenging capacity (PSC)

Total antioxidant activities measured by peroxyl radical scavenging capacity (PSC) for the 24 cultivars are presented in Table 1with the PSC values expressed as mg of vitamin C equivalents/100 g FW. Royalty had the highest PSC values, and was significantly higher than other cultivars (*P*<0.05). Corbeau, Dornfelder and the other red flesh cultivar Salvador also had high PSC values (*P*<0.05). PSC values of all green yellow cultivars were lower than 100 mg of vitamin C equivalents/100 g FW except for Melon. In addition, most PSC values of colored cultivars were higher than 109 mg of vitamin C equivalents/100 g FW except for a red cultivar (Agwam) and a blue black cultivar (Ribier). Dornfelder had the highest PSC value in all table grapes, followed by Demir Kara, Yourutico, Pirobelle and Mathidle. The other PSC values of table grapes were no more than 73 mg of vitamin C equivalents/100 g FW. Most wine grapes had high PSC values except for Agwam, Robola and Melon.

### Cellular antioxidant activities

CAA values for 24 grape cultivars using the no PBS wash protocol are shown in Table 1. CAA values ranged from 3.9 to 139.9 *µ*mol of QE/100 g of grape, and the mean was 41.5 *µ*mol of QE/100 g of grape. Royalty had the highest CAA value (*P*<0.05), followed by Salvador and Ruby Cabernet (*P*<0.05). Demir Kara, Dornfelder, Touriga, Saint Macaire, Mermark, Robola, Corbeau and Yourutico, Cabernet Sauvignon, Melon had intermediate high CAA activities, but were not significantly different from each other (*P*>0.05). Then the rankings of CAA activities were followed by Plavac Mali, Araclinos, Pearl of Zola, Pirobelle, Ribier and Coudsi. Madam Matijas, Thompson Seedless, Mathilde, Agwam and Muscat Alexandria had the lowest CAA values among the 24 grape cultivars tested.

In using the PBS wash protocol, CAA values ranged from 1.4to 95.8 *µ*mol of QE/100 g of grape, and the mean was 25.7 *µ*mol of QE/100 g of grape (Table 2). Royalty had the greatest cellular antioxidant activity among the cultivars tested (*P*<0.05). Salvador ranked second, and Ruby Cabernet and Dornfelder were third and fourth in CAA activities, respectively (*P*<0.05). Plavac Mali and Corbeau and other cultivars were in descending order in their CAA activities. Muscat of Alexandria and Agwam had the lowest CAA values. In both PBS wash and no PBS wash protocols, the CAA values were significantly higher in wine grapes than in table grapes. This was also true in colored grapes when compared with green-yellow grapes (*P*<0.05, Table 2).

**Table 2:** Mean contents of total phenolics, total flavonoids, ORAC and PSC values among different types of grapes (mean±SD).

	Primary use		Color type	
	Table grape	Wine grape	Colored grape	Green yellow grape
Total phenolics (GAE/100 g)	250.8±31.6^b^ *	444.6±42.0^a^	399.3±37.7^a^	250.0±51.4^b^
Total flavonoids (CE/100 g)	264.8±44.0^b^	526.3±64.2^a^	462.8±57.4^a^	269.6±65.8^b^
ORAC values (TE/100 g)	830.6±122.0^b^	1769.0 ±206.1^a^	1553.0 ±186.4^a^	818.6±181.4^b^
PSC values (VE/100 g)	107.4±33.2^b^	248.7±39.1^a^	236.4±33.8^a^	56.5±13.1^b^
CAA with PBS wash (QE/100 g)	17.5±12.9^b^	31.6±29.2^a^	32.0±26.8^a^	10.3±4.8^b^
CAA with no PBS wash (QE/100 g)	27.7±18.4^b^	51.4±33.8^a^	49.3±31.8^a^	22.5±16.0^b^

Note: GAE, CE, TE and VE represent mg of gallic acid, catechin, Trolox and vitamin C equivalent respectively; QE represents µmol of quercetin equivalent.

*Values with no letters in common in each row are significantly different (*P*<0.05).

### Correlationsamong total phenolics, total flavonoids and antioxidant activities

Correlations among the total phenolics, total flavonoids, ORAC values and PSC values were analyzed (Table 3). Significantly positive correlations were found between total phenolics and total flavonoids (*r* ^2^ = 0.888), and between ORAC and PSC values (*r* ^2^ = 0.863). The ORAC values had significantly positive correlations with total phenolics (*r* ^2^ = 0.924) and total flavonoids (*r^2^* = 0.862). In comparison, the PSC values also had significantly positive correlations with total phenolics (*r* ^2^ = 0.757) and total flavonoids (*r* ^2^ = 0.737), but the correlation coefficients were lower than those observed for the ORAC values.

**Table 3:** The relationship among total phenolics, total flavonoids, ORAC, PSC and CAA values.

	Total phenolics	Total flavonoids	ORAC values	PSC values
Total phenolics	1			
Total flavonoids	0.888*			
ORAC values	0.924	0.862		
PSC values	0.757	0.737	0.853	
CAA values(no PBS wash)	0.788	0.848	0.899	0.809
CAA values(PBS wash)	0.574	0.741	0.736	0.739

*All correlation coefficients were significant at *P*<0.01.

The relationships of total phenolics content, total flavonoids content, ORAC values, and PSC values with CAA values were also determined. CAA values were significantly positively correlated with total phenolics (*r* ^2^ = 0.574), total flavonoids (*r* ^2^ = 0.741), ORAC values (*r* ^2^ = 0.736) and PSC values (*r* ^2^ = 0.739) when no PBS wash protocol was used (*P*<0.05). While significantly positive correlation relationships were also found between CAA values and total phenolics, total flavonoids, ORAC values and PSC values using the PBS wash protocol (*P*<0.05),the coefficients of correlation were lower than those with no PBS wash.

### Inhibition of human cancer cell proliferation

**Figure 1:**
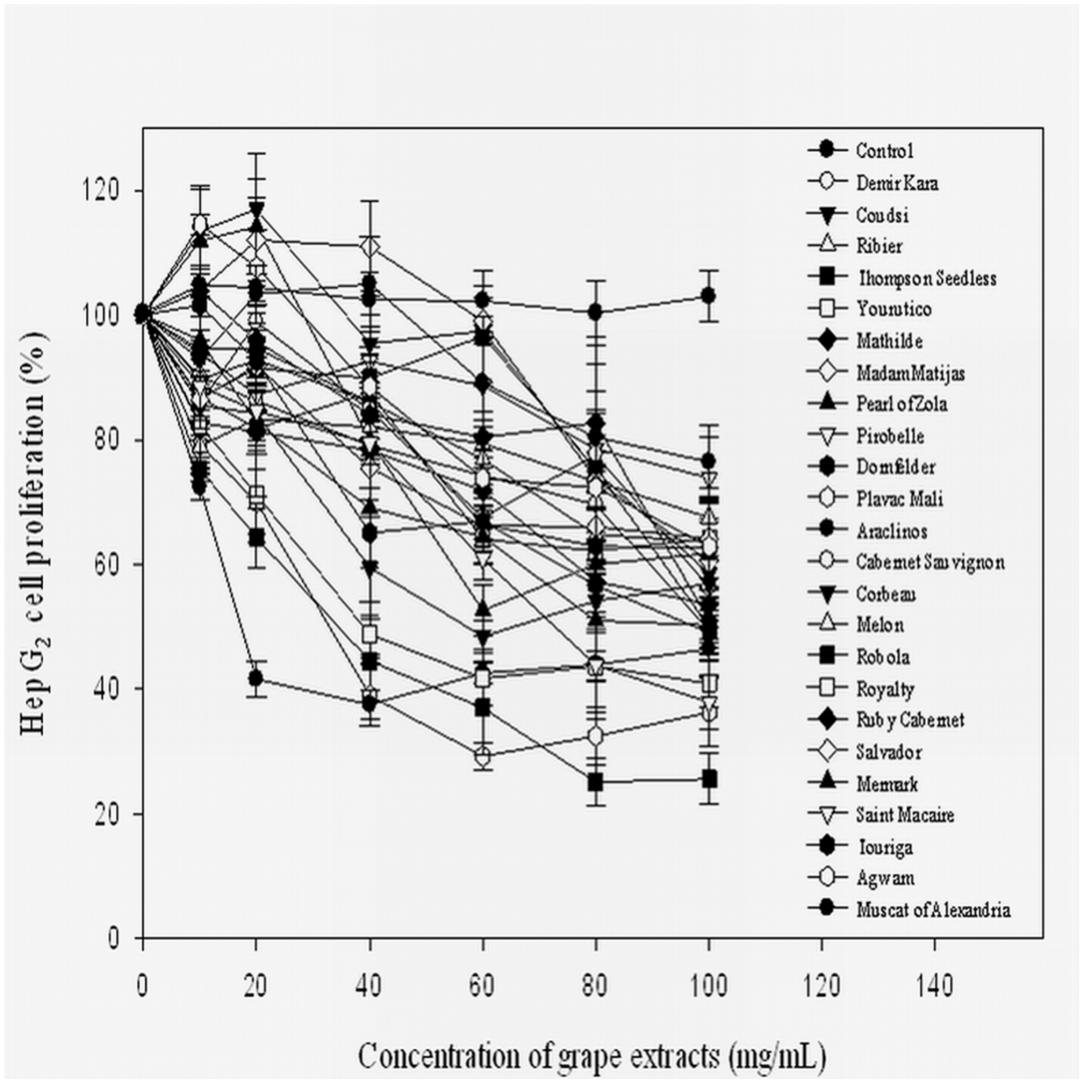
Inhibition of HepG2 cell proliferation by 24 grape extracts.

The effects of extracts from the 24 grape cultivars against the growth of HepG2 human liver cancer cells *in vitro* are presented in Figure 1. Overall, grape extracts had potent antiproliferative activities against HepG2 human liver cancer cell growth in dose-dependent manners. The inhibition of cancer cell proliferation by the 24 grape extracts ranged from 25 to 82% at the concentration of 100 mg/mL extract applied (Figure 1). Robola, Plavac Mali, Royalty, Saint Macaire, Touriga, Coudsi, Ruby Cabernet,and Demir Kara exhibited relatively higher antiproliferative activities towards HepG_2_ cells (Figure 1and Table 4). The EC_25_ and EC_50_of the antiproliferative activities of different grape cultivars are presented in Table 4. Lower EC_25_ and EC_50_values represent higher antiproliferative activities. There were no significant differences in antiproliferative activities between wine and table grapes, but in general, the antiproliferative activities of red grapes were higher than those of yellow-green grapes. There were no detected cytotoxicities towards HepG2 cells *in vitro* at the concentration of 150 mg/mL of grape extracts in all 24 grape cultivars tested, indicating that antiproliferative activities were not caused by cytoxicity [32].

**Table 4:** Antiproliferative activities (EC_25_ and EC_50_) and cytotoxicities (CC_50_) of grape extracts towards human HepG2 liver cancer cells.

Cultivar	Antiproliferative Activity	
	EC_25_ (mg/mL)	EC_50_ (mg/mL)
Araclinos	1.53±0.25	ND*
Robola	10.40±1.41	33.63±4.04
Plavac Mali	11.57±2.43	38.70±11.73
Royalty	14.50±1.57	54.97±2.40
Corbeau	25.43±4.40	ND
Mathilde	30.83±11.17	ND
Saint Macaire	31.13±2.81	79.90±13.14
Mermark	39.43±2.82	ND
Pearl of Zola	39.97±2.17	ND
Demir Kara	42.40±3.76	138.23±10.17
Touriga	43.90±1.40	97.00±6.53
Salvador	48.30±8.02	ND
Yourutico	49.50±6.15	ND
Cabernet Sauvignon	55.80±4.90	ND
Ribier	66.07±6.54	ND
Dornfelder	66.50±6.99	ND
Ruby Cabernet	68.93±3.16	132.80±3.03
Agwam	71.47±2.34	ND
Melon	89.10±1.14	ND
Coudsi	90.80±4.74	129.40±2.91
Madam Matijas	95.67±2.30	ND
Thompson Seedless	101.27±3.30	ND
Pirobelle	108.00±16.81	ND
Muscat of Alexandria	112.77±6.52	ND

*ND, not detected, CC50>150 mg/mL.

## Discussion

Regular consumption of fruits and vegetables has been linked to reduced risk of developing chronic diseases [33], [34]. Grapes and their products are rich in phytochemicals, which are proven to possess many health benefits. Grape phytochemicals include anthocyanins, flavanols, flavonols, stilbenes (resveratrol) and phenolic acids and their contents and profiles can vary significantly among different *V. vinifera* cultivars [2], [35].There have been studies attempting to link phytochemical profiles with their total antioxidant activities in *V*. *vinifera*, but these studies were limited to just a few cultivars [4]. Here we reported phytochemical profiles of 24 *V. vinifera* cultivars and studied the relationships among total phenolics, total flavonoids, total antioxidant activities, cellular antioxidant activities, and antiproliferative activities in these cultivars. These 24 *V. vinifera* cultivars were a part of the core collection of *V. vinifera* germplasm preserved at the USDA-ARS *Vitis* clonal repository in Davis, California and they were selected for this study on the basis of their compositions and contents of polyphenolic compounds reported in a previous study [2].

The present study, using a different, more accurate detection method for the total phenolic compounds, confirmed our previous result that the 24 cultivars selected for this study had a wide range of variation in their total phenolic contents. The mean total phenolic content ranged from 95.3 (Muscat of Alexandria) to 686.5 (Royalty) mg of gallic acid equivalents/100 g FW. In contrast, Yang et al. [4] reported that the total phenolic contents of 14 wine grapes ranged from 201.1 to 424.6 mg of gallic acid equivalent/100 g of fruits. The difference in these observations, to a large extent, was certainly attributed to different cultivars used in the studies. It should be pointed out that there could be some other factors which may contribute to these differences. For example, samples collected from different vineyards or vintages could have significant influence on the total phenolic content as reported by Arnous et al [36] and Kallithraka et al. [37]. The wide range of variation of total phenolic contents among the grape cultivars in this study offered us a unique opportunity to investigate the correlative relationships of phenolic compounds with some health benefit measurements such as antioxidant and antiproliferative activities. In consistence with our earlier study [2], the total phenolic contents of wine grapes were significantly higher than those of table grapes (*P*<0.05); and total phenolics of colored grape cultivars were higher than those of green yellow grape cultivars (*P*<0.05, Table 2).

This study was the first to determine total flavonoid content in grapes using a new method developed [38]. Determination of total flavonoids in foods is challenging. The most common methods for determining the content of total flavonoids include Aluminum Chloride (AlCl_3_) colorimetric assay [4], [38] and high performance liquid chromatography (HPLC). However, these two methods each have certain limitations. AlCl_3_ colorimetric assay only measures partial flavonoids and therefore cannot be used accurately to determine total flavonoids [38], [39]. HPLC is an excellent method to determine individual flavonoids, but cannot be used to determine the total flavonoids because the method is limited by the availability of flavonoid standards and many un-identified flavonoids are present in foods [33], [38]. The SBC assay developed by our lab can detect all types of flavonoids, including flavones, flavonols, flavonones, flavononols, isoflavonoids and anthocyanins [38]. The total flavonoid contents in the 24 grape cultivars, measured by SBC assay varied from 94.7 to 1055 mg of catechin equivalents/100 g FW. Anthocyanins were the dominant in total flavonoids in colored grape cultivars according to our previous study [2]. The total flavonoid contents of wine grapes were significantly higher than those of table grapes (*P*<0.05); and total flavonoids of colored grape cultivars were higher than those of green yellow grape cultivars (*P*<0.05, Table 2) because colored grape cultivars had high anthocyanins content and most wine grape cultivars were colored grapes. These results were consistent with those reported as the sum of individual flavonoids using HPLC [2].

The contribution of total flavonoids to total phenolics, calculated on the micromole basis, ranged from 40.3 to 91.3%. Further analysis found no significant differences in the contributions of total flavonoids to total phenolics between table and wine grapes, or between colored and green yellow grapes. These results suggested that flavonoids are one of the major phytochemicals in grapes and their relative contributions to the total phenolics are not dependent on the types of grape cultivars (i.e. wine vs. table grapes).

The mean ORAC values of wine grapes were 1769±206.1 mg of Trolox equivalents/100 g FW and significantly higher than those in table grapes (*P*<0.05, Table 2). In addition, the ORAC values of colored grapes were significantly higher than those of green yellow grapes (*P*<0.05, Table 2).

The PSC values for the 24 grape cultivars ranged from14.2 (Muscat of Alexandria) to 557 (Royalty) mg of vitamin C equivalents/100 g FW, indicating that there was a wide-range of variation across all 24 grape cultivars. Adom and Liu [24] reported the PSC value of red grapes was 371 mg of vitamin C equivalents/100 g FW, which was in the range of the results we reported here. The mean of PSC values of wine grapes was significantly higher than that of table grapes (*P*<0.05, Table 2). Similarly, the PSC values of colored grape cultivars were higher than those of green yellow grape cultivars (Table 2).

The 24 cultivars were also evaluated for their antioxidant activity in the CAA assay. The CAA values of Royalty, Salvador, Ruby Cabernet, Demir Kara, Dornfelder and Corbeauwere higher than other cultivars (Figure 1). These cultivars also had higher total phenolics and total flavonoids, and higher ORAC and PSC values. Among these cultivars, Royalty, Salvador and Corbeau are red flesh grape cultivars and tend to be rich in anthocyanins. The mean CAA values were 32.0 *µ*mol of QE/100 g of grape for colored grape cultivars and 10.3 *µ*mol for green yellow grape cultivars using the PBS wash protocol. But higher CAA values were observed when no PBS wash protocol was used (49.3 *µ*mol of QE/100 g of grape for colored grape cultivars and 22.5 *µ*mol for green yellow grape cultivars). We also observed that the CAA values in wine grapes were significantly higher than these in table grapes. The general trends of these results were in agreement with those of Wolfe et al. [29], [30], but this study covered a much larger range of cultivar variation in terms of both color and end-product types.

The inhibition of HepG2 liver cancer cell proliferation by extracts from the 24 grape cultivars ranged from 25 to 82% at the concentration of 100 mg/mL extracts applied (Figure 1). Yang et al. [4] reported that the phytochemical extracts of Pinot Noir and Chardonnay varieties exhibited strong antiproliferative activity towards HepG2 cells with the lowest EC_50_values of 17.0±0.8 mg/mL and 18.1±0.1 mg/mL (*p*<0.05), respectively. On the other hand, the phytochemical extracts of Vidal Blanc and DeChaunac showed a relatively weak antiproliferative activity with higher EC_50_values of 52.1±2.1 and 52.2±3.0 mg/mL, respectively [4]. Our results in this study were similar to the results of Vidal Blanc and DeChaunacas reported by Yang et al. [4]. In consistent with other results, the colored grape cultivars had higher antiproliferative activities than those of green yellow grape cultivars.

Correlations among total phenolics, total flavonoids, total antioxidant activity, cell proliferation, and cellular antiocxdant activities were analyzed. There were significantly correlative relationship among total phenolics, total flavonoids and antioxidant activities, which were in agreement with what was reported by Arnous et al [36] and Kallithraka et al. [37]. No significantly correlative relationships were found between total antioxidant and antiproliferative activities against HepG2 liver cancer cells (*P*>0.05). Additionally, the total phenolic and flavonoid contents of grapes did not correlate with the antiproliferative activities. These results were consistent with what were previously reported in literature [17], [39], suggesting that specific phytochemicals, rather than the total phytochemicals, might be responsible for the antiproliferative activities. Different phytochemicals might act additively and/or synergistically to contribute to the total antiproliferative activities in grapes. The CAA values for fruits were significantly positively related to total phenolic content and total flavonoids when log-transformed data were analyzed (*P*<0.05)in this study. The correlation coefficients for CAA values and total phenolics, total flavonoids, ORAC and PSC values were higher for the no PBS wash protocol than these for the PBS wash protocol in this study. These results were consistent with what Wolfe et al. reported previously [23]. This is in contrast to a study involving vegetable extracts, in which prevention of DCFH oxidation in HepG2 cells by vegetable extracts was not correlated to ORAC or total phenolics [40]. Our current study suggested that total phenolic content might be a better predictor for the cellular antioxidant activity of grapes than the ORAC value, despite the commonality of measuring peroxyl radical scavenging abilities in both of the antioxidant activity assays.

Among the 24 cultivars evaluated, Royalty grape showed highest total phenolic content, total flavonoid content, ORAC, PSC and CAA values. It was the one which had consistent and high correlations among these measurements. Several other cultivars such as Corbeau, Salvador and Mermark also had high levels of phytochemicals, and antioxidant activities. On the other hand, Cabernet Sauvignon, a well-known wine grape cultivar, had relative high levels of total phenolic and flavonoid contents, but its ORAC and PSC values were about average. Some of the differences are certainly due to varietal genetic background. For example, Royalty is a red flesh cultivar with abundant anthocyanins produced in whole fruits, while many other cultivars including Cabernet Sauvignon are not.

In summary, grapes are rich in phenolics and flavonoids. Although viticulture practices, environmental conditions, and post-harvest processing can affect the content of total phenolics, total flavonoids, or individual bioactive compounds in grapes and grape products, varietal or genetic difference is one of the most important factors. Here we observed tremendous variation in total phenolic and total flavonoid contents, and ORAC, PSC and CAA values among the24 grape cultivars tested. The total antioxidant activities of grapes were significantly correlated with the total phenolics and flavonoids. However, no significant correlations were found between total phenolic content, total flavonoid content, ORAC, PSC values, or CAA values with the antiproliferative activities in this study. Lack of such correlations provides both challenges and opportunities in future studies. Total phenolics, total flavonoids, ORAC, PSC and CAA values of wine grapes and colored grapes were significantly higher than those of table grapes and green-yellow grapes. Another important finding from this study was the identification of several germplasm accessions with much high contents of phenolics and flavonoids, and total antioxidant activity. This information is important for breeding grape cultivars with better nutritional qualities of wine and table grapes in the future.
